# Factors contributing to transient-induced fading: Examining the impact of luminance contrasts and subjective contours

**DOI:** 10.1177/20416695241290462

**Published:** 2024-10-30

**Authors:** Moyou Jiang, Hiroyuki Ito, Tama Kanematsu

**Affiliations:** 12923 Kyushu University, Japan

**Keywords:** transient-induced fading, filling-in, Troxler fading, Kanizsa-type subjective contour

## Abstract

Transient-induced fading is a phenomenon where a peripheral target perceptually fades when a surrounding object is flashed. It has been suggested that the transient-induced fading could be affected not only by the lower-level factors such as the luminance contrast change, but also by the higher-level factors such as Gestalt grouping by similarity. In the present study, Experiment 1 investigated whether the perceptual fading of a visual target could be strongly induced when a ring area surrounding the target with high luminance contrast disappeared rather than appeared. Experiment 2 examined the effect of the (dis)appearance of a higher-level object (Kanizsa-type subjective contour) on the fading perception. Experiment 3 further investigated whether the rating of the perceived effortlessness of a subjective contour could be positively correlated with the fading duration of the target. Our results revealed that perceptual fading was mainly induced by the disappearance of fan areas inside black disks producing a subjective contour surrounding the target. Disappearance of a perceptual object at the representation level does not trigger the transient-induced fading even if a higher-level factor (e.g., grouping by similarity) affects the fading objects.

There is often a discrepancy between the detection of an image on the retina and the perception of it. In the blind spot where the image of a visual target cannot be captured on the retina, a phenomenon called filling-in occurs, in which the brain appropriately fills in the blind area based on the surrounding information (Durgin et al., 1995; Paradiso & Nakayama, 1991; Ramachandran, 1992; Spillmann et al., 2006; Weil & Rees, 2011). Because of the filling-in process, people believe they see their entire visual field in daily life without noticing any missing visual images on the retina. A phenomenon similar to the filling-in of the blind spot also occurs in retinal scotomas (Zur & Ullman, 2003).

On the other hand, many visual masking studies have previously shown that even when the image of a visual target is detected on the retina, its visibility can be reduced by a temporally successive stimulus, called the mask (Bachmann, 1994; Bachmann & Francis, 2013; Breitmeyer & Ogmen, 2006; Breitmeyer, 1984; Breitmeyer & Ogmen, 2000). When the mask appears before the target stimulus presentation, forward masking is induced (also known as paracontrast masking, see Ogmen et al., 2003); while backward masking is induced if the target is presented first, followed by the mask (also known as metacontrast masking, see Fry, 1934; Ogmen et al., 2003). As a similar procedure to the metacontrast masking, Breitmeyer and Rudd (1981) proposed a single-transient masking paradigm, in which the visual target indicated as a vertical black bar perceptually disappeared when a brief, transient 50 ms stripe was presented 2 s after the target onset at the left/right side of the target. Similarly, Kanai and Kamitani (2003) reported a transient-induced fading phenomenon in which the target stimulus indicated by a red disk perceptually disappeared when a white ring was flashed around the target for 40 ms against a near-isoluminant green background. In the transient-induced fading, a visual target would perceptually disappear in a time-locked way at the moment a transient (flash) stimulus is presented around it (also see Kanai et al., 2005; Moradi & Shimojo, 2004). Another type of perceptual fading, which arises spontaneously with steady fixation for several seconds, has also been reported. For example, in Troxler fading, when an observer steadily focuses on the fixation point, visual stimuli presented peripherally would gradually fade and then completely disappear (Troxler, 1804). Some previous studies attribute Troxler fading to local adaptation to luminance edges (Ramachandran & Gregory, 1991; Vergeer & van Lier, 2007).

The perceptual fading and the filling-in noted above may appear to be opposing phenomena. However, because the area where an object faded away appears to be filled with the surrounding information in both effects, they could be considered a single phenomenon. When Troxler fading occurs, the retinal area of the visual target is filled in by the background color. In Kanai and Kamitani (2003), the green color of the background seemed to fill in the area of the red target when transient-induced fading occurred. The color of the target area perceptually became the same color as the background, neither black nor “empty.” The previous studies on perceptual fading seem to show that perceptual fading of an object occurs, accompanied by the filling-in of the background (Anstis, 2013; Bonneh et al., 2010; Kanai & Kamitani, 2003; May et al., 2003; Pritchard, 1961; Troxler, 1804).

The role of transients in a perceptual fading phenomenon has also been investigated. Simons et al. (2006) reported that a sudden contrast decrement led a blurred low-contrast photograph of a scene to fade into a plain image with uniform luminance and hue. In the second experiment of Simons et al. (2006), they presented photographs of natural scenes with or without static black disks superimposed on top of the scene in a 15 s trial. In some conditions, they added or removed the static black disks after the first 10 s of the trial. They found that when the static black disks disappeared abruptly, the entire scene was induced to fade more greatly than when the black disks appeared abruptly.

The purpose of the present paper is to investigate what triggers the transient-induced fading of an object. The above-noted studies employed a high-contrast luminance-defined object as a fading inducer. It has been shown that the flash or (dis)appearance of high-contrast objects induces perceptual fading. However, it is not clear whether the change in luminance contrast in the scene or (dis)appearance of objects is important to trigger the transient induced fading. Actually, there is a study that shows the involvement of high-level object representation with transient-induced fading. Vergeer and van Lier (2007) proposed that when the targets presented below the fixation point were similar in color (red or green) or in shape (circle or triangle), a flash stimulus presented around one of them induced them to jointly fade more than when they were in different colors or shapes. This implies that the higher-level factors such as grouping by similarity have some impact on the transient-induced fading.

It has been known that perceptual grouping usually serves to consolidate individual local elements into coherent patterns such as object representations (Murray et al., 2004; Razpurker-Apfeld & Pratt, 2008; Wang et al., 2012). This is especially so in Kanizsa-type subjective contours (Kanizsa, 1955), which have been shown to function as a visual “object” in psychophysical experiments of apparent motion (Mather, 1988; Ramachandran, 1985), stereo capture (Ramachandran & Cavanagh, 1985), visual search (Davis & Driver, 1994), depth perception (Sato, 1984), infant vision (Kavšnek & Yonas, 2006), fish vision (Sovrano & Bisazza, 2009), or the visual saltation illusion (Ito et al., 2023). A variety of studies also showed that perceptual grouping is closely related to the process of surface segmentation which could lead to an integrated, partial object representation (Bakar et al., 2008; Stanley & Rubin, 2003; Wang et al., 2012).

In the present paper, we used Kanizsa-type subjective contours to separate the appearance and disappearance of a perceptual object from those of local luminance contrasts. As shown in Figure 1(A), when the three local sectors (fan-shaped parts of black disks) disappear, a subjective surface/visual object (subjective triangle here) partially occluding three black disks appears. As shown in Figure 1(B), oppositely, when the sectors appear, the subjective object perceptually disappears. That is, the appearance of the Kanizsa-type subjective figure was at the same time the disappearance of the three black fan areas, and vice versa. In perceptual fading due to transients, the disappearance of fading inducers is considered as a stronger trigger than their appearance (Kanai & Kamitani, 2003; Simons et al., 2006). Then we investigated whether the partial disappearance of luminance contrasts or the perceptual disappearance of the subjective contour caused the transient-induced fading more effectively. If the disappearance of the subjective figure is more effective in inducing the perceptual fading of a targeted object than its appearance, we could consider that the disappearance in the object representation level should be important for the transient-induced fading. In Experiment 1, we investigated the difference in the induced fading effect between the appearance and disappearance of high-contrast fading inducers. Experiment 2 then investigated whether the (dis)appearance of a perceptual object affects the fading phenomenon using a Kanizsa-type subjective contour. In Experiment 3, we examined whether the perception of a subjective contour was an important factor in eliciting a strong transient-induced fading phenomenon.

**Figure 1.**
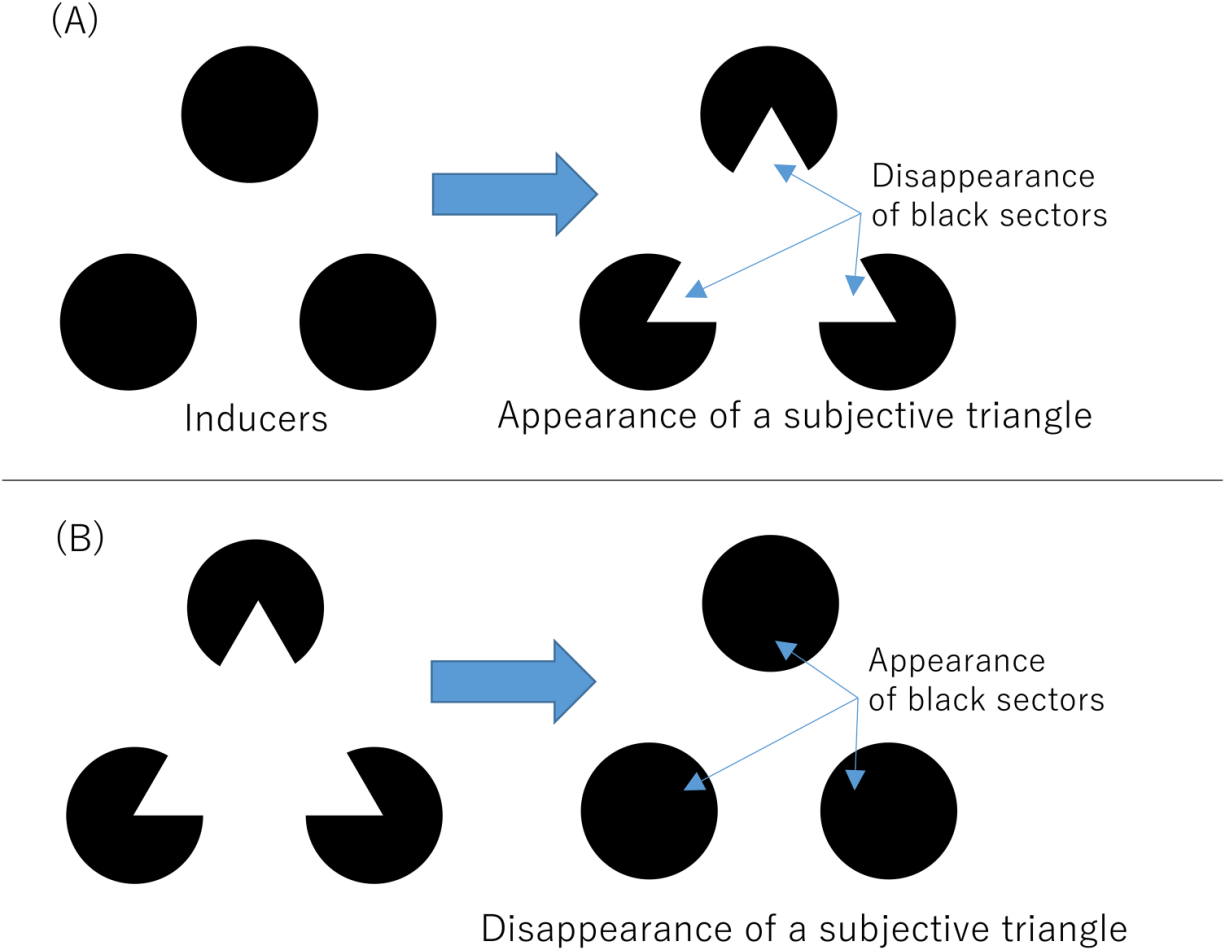
Appearance and disappearance of the Kanizsa triangle. (A) shows the appearance of a subjective triangle with disappearance of black sectors. (B) shows the disappearance of a subjective triangle with appearance of black sectors.

## Experiment 1

Experiment 1 examined whether the (dis)appearance of luminance contrasts could have an impact on transient-induced fading perception with a stimulus configuration used in Kanai and Kamitani (2003).

### Methods

#### Participants

Twenty-two graduate students (8 males and 14 females) from Kyushu University, participated in this experiment. One female participant withdrew from the experiment due to visual fatigue. Their ages ranged from 22 to 31 years old (25.1 ± 2.2 years). All participants had normal or corrected-to-normal vision and were naive to the purpose of the study. They were informed prior to the experiment that their privacy was guaranteed and written informed consent was obtained from all participants. The study was approved by the local ethics committee of Kyushu University.

#### Apparatus

The experiment was conducted in a darkened room. Visual stimuli were generated in real-time on a computer (Apple, MacBook Air Liquid Retina) and presented on a display (GIGABYTE, G24F 2 Gaming Monitor). The screen of this display was treated as a matrix of 1920 × 1080 pixels. The refresh rate was 60 Hz.

#### Stimuli

**Figure 2.**
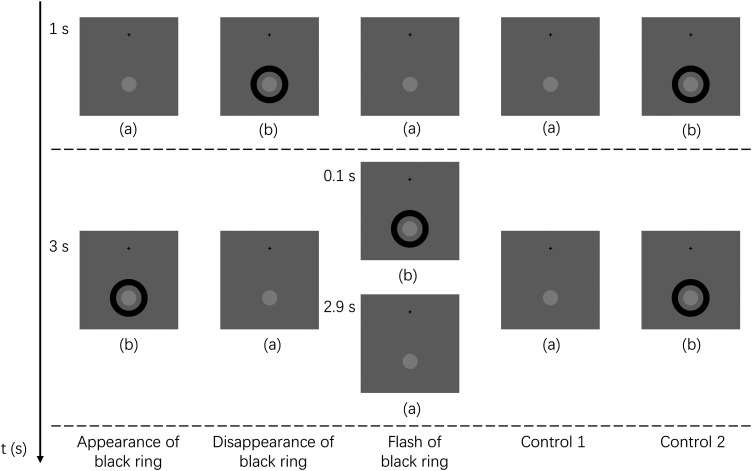
The visual stimuli as well as the presentation of the five stimulus conditions in experiment 1. The black cross and the target indicated by the light gray disk were always presented for 4.0 s throughout all the trials under all conditions. (a) shows the stimulus image where a fixation cross and a target (i.e., light gray disk) were presented. (b) shows the stimulus image where a fixation cross, a target, and a fade inducer (i.e., black ring) were presented. In the *appearance of black ring* condition, the black ring was presented one second after the start of the trial and displayed for three seconds. In the *disappearance of black ring* condition, the black ring was presented at the same time as the start of the trial and disappeared after one second. In the *flash of black ring* condition, the black ring was presented just for 0.1 s, one second after the start of the trial. For the control conditions, the black ring was either never presented in the trial (*control 1*), or always present in the trial (*control 2*).

There were five stimulus conditions in Experiment 1 (see Figure 2). Throughout all stimulus presentations, a black fixation cross was always presented at the center of the screen. The target (i.e., the light gray disk) was always presented against the gray background (75.6 cd/m^2^ in luminance) at the bottom peripheral area at an angular eccentricity of 9.0° from the fixation point (Figure 2(a)). In visual angle, the diameter of the target was 2.6°. For the black ring, the diameter was 6.8° from the outer border and 4.6° from the inner border (Figure 2(b)). The center point was shared by the target and the black ring. The luminance of the target was measured as 94.4 cd/m^2^ and the black ring was 1.43 cd/m^2^.

#### Procedure

The participants observed the display at a viewing distance of 57 cm using a chinrest. The experimenter first explained the definition of the perceptual fading phenomenon, then the participants performed a practice session to verify they understood the illusion. They were instructed to focus on the black fixation cross in the center of the screen and not to blink throughout each trial (4.0 s). During the observation, the participants were instructed to press the space key of the keyboard when they felt the target completely disappeared and release the space key only when the target reappeared or when the trial ended. After 4.0 s presentation, a noise stimulus was presented for 6 s to eliminate the afterimage effect. In Experiment 1, five conditions were randomly presented in one block. Each block was repeated 10 times. Thus, a total of 50 trials (5 conditions × 10 repetitions) were conducted in Experiment 1. Between every two blocks, the participants were required to rest at least 1 min to eliminate visual fatigue. After resting, they started a new block at their convenience.

### Results and Discussion

**Figure 3.**
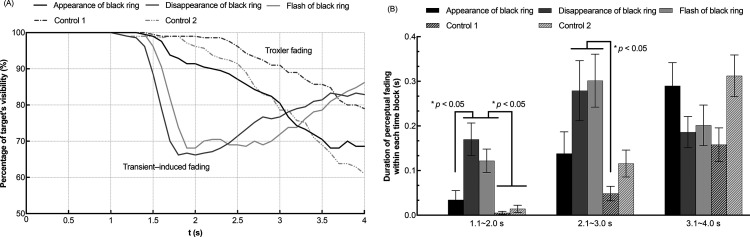
Results of experiment 1. (A) shows the time course results of the target's fading perception. The horizontal axis indicates the time elapsed after stimulus presentation in each trial. The vertical axis indicates the percentage of the target's visibility. (B) shows the time-blocked results of the target's fading duration in each trial. The horizontal axis indicates the divided three blocks of time course (1.1 ∼ 2.0 s, 2.1 ∼ 3.0 s, and 3.1 ∼ 4.0 s). The vertical axis indicates the duration of the target's fading within each time block. Error bars indicate standard errors of the means (SEs).

The results of Experiment 1 are shown in Figure 3. Specifically, Figure 3(A) depicts the time course results showing the percentage of target's visibility at each time point as time passed; Figure 3(B) depicts the time-blocked results showing the duration of target's perceptual fading within each time block.

To quantitatively analyze the results, the key status (pressed or released) was sampled with 0.1 s time intervals. The value of “1” was assigned if the “space” key was pressed or held down and “0” if no key was pressed during the interval. To acquire the percentage of target's visibility, we first calculated each participant's and each condition's percentage of “0” responses at each time point across 10 trials. This value represented the percentage of the target's visibility at that time point. Second, the individually acquired percentages were averaged across the 21 participants at each of 40 time points (0.1 s ∼ 4.0 s) and plotted separately for each condition in Figure 3(A).

To conduct a statistical analysis, the time course of each trial after 1.0 s of presentation was divided into three time blocks: 1.1 ∼ 2.0 s, 2.1 ∼ 3.0 s, and 3.1 ∼ 4.0 s. To acquire the duration of the target's perceptual fading within each time block, the frequency of “1” out of 10 key responses for each time interval sampled within each time block in one trial was obtained for each participant and each condition. The frequency value was then multiplied by 0.1 s and averaged across 10 trials. The individually calculated durations of the target's perceptual fading within each time block were averaged across all participants and shown in Figure 3(B) separately for each time block and condition.

As shown in Figure 3(A), from 0.1 s to 1.0 s, the target did not fade under all conditions. In the *disappearance of black ring* condition, the target was perceived to fade suddenly at around 1.3 s and then gradually became visible again after around 2.0 s. Similarly, in the *flash of black ring* condition, the target was perceived to fade suddenly at around 1.5 s and then gradually became visible again after around 2.5 s. When reaction times are taken into account, these fadings may have been triggered by the transient at 1.0 s. The other three conditions showed a similar tendency that the target gradually faded as time passed. This clear difference among the conditions in time course may indicate that the Troxler fading phenomenon was mainly induced in the *appearance of black ring* and two *control* conditions, while the transient-induced fading mainly arose in the *disappearance of black ring* and the *flash of black ring* conditions.

As shown in Figure 3(B), two-way analysis of variance (ANOVA) revealed that the main effect of the black ring's presentation type (*appearance, disappearance, flash, control 1,* and *control 2*) was significant for the target visibility [*F*_4, 80 _= 4.499, *p *= .002, η_p_^2 ^= .184]. The main effect of the time block was also significant for the target visibility [*F*_2, 40 _= 20.541, *p *< .001, η_p_^2 ^= .507]. The interaction between the two independent variables was also significant [*F*_8, 160 _= 9.376, *p *< .001, η_p_^2 ^= .319]. Specifically, the simple main effect analysis with Bonferroni correction showed that in the presentation time block of 1.1 ∼ 2.0 s, the target's fading was induced longer in the *disappearance of black ring* and *flash of black ring* conditions than in the *appearance of black ring* and two *control* conditions (*p *< .05). In the presentation time block of 2.1 ∼ 3.0 s, target's fading was induced longer in the *disappearance of black ring* and *flash of black ring* conditions than the *control-1* condition (*p *< .05).

These results indicated that the *disappearance of black ring* and *flash of black ring* conditions induced a quicker and stronger transient-induced fading perception than the other three conditions. Thus, we confirmed that the transient-induced fading was stronger when the luminance-defined object around the target disappeared rather than when it appeared. However, from the results, it was not clear whether the strong fading effect was caused by the disappearance in perceptual representation of a visual object or by the luminance contrast decrement at the ring area. Experiment 2 investigated this point with the Kanizsa triangle.

## Experiment 2

In order to investigate the change in luminance and higher-level factors separately, we used a Kanizsa triangle display and modulated three black disks around the target (see Figure 1). As shown in Figure 1(A), when the three sector parts oriented toward the target disappeared, a subjective triangle appeared as a perceptual object on the top of the black disks, and vice versa as in Figure 1(B). Experiment 2 then investigated whether the disappearance of a perceptual object (subjective triangle) could have an impact on transient-induced fading perception. If the disappearance of the luminance contrast is dominant in inducing the transient induced fading, the appearance of the subjective triangle would dominantly produce the perceptual fading rather than its disappearance.

### Methods

#### Participants

The participants were the same as in Experiment 1.

#### Apparatus

The apparatus used in Experiment 2 was the same as Experiment 1.

#### Stimuli

**Figure 4.**
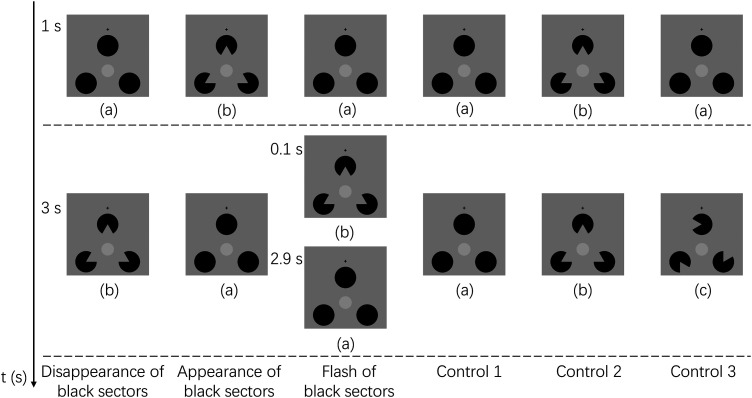
The visual stimuli as well as the presentation of the six stimulus conditions in experiment 2. The black cross and the target were always presented for 4.0 s throughout all trials. Stimulus images depict the target with (a) three black disks, (b) missing sectors forming a subjective “triangle,” and (c) three notched circles with their angles not oriented toward the target. After one second, the sectors inside black disks would disappear, appear for 3.0 s, or flash for just 0.1 s, for the *disappearance of black sectors, appearance of black sectors or flash of black sectors* conditions, respectively. For the three control conditions, the three black sectors either never disappeared (*control 1*), were presented toward the target throughout the trial (Kanizsa triangle was continuously displayed) (*control 2*), or disappeared toward different orientations after 1 s (*control 3*).

There were six stimulus conditions in Experiment 2 (see Figure 4). The fixation cross and target (light gray disk) as well as the gray background remained the same as Experiment 1. The luminance of the target was 94.7 cd/m^2^ and the background was 75.7 cd/m^2^. Instead of a black ring, Experiment 2 used three black disks with one presented above the target and two presented at the left/right side of the target as shown in Figure 4(a). The center of the top black disk was vertically aligned with the target's center. For the left and right disks, their centers were at the same horizontal level below the target's center. The distance from the target's center to each black disk's center was the same, being 5.5°. This way, the centers of the three black disks could be considered as vertices of an equilateral triangle. The diameter of each black disk subtended a visual angle of 4.4°. The luminance of each black disk was 1.44 cd/m^2^. Specifically, when the angles of the three sectors were 60° in each black disk and when they simultaneously disappeared toward the center of the target, it would be perceived as a subjective triangle as shown in Figure 4(b). In contrast, when the missing sectors in Figure 4(b) simultaneously appeared, forming Figure 4(a), the subjective triangle as a visual object perceptually vanished. For the subjective triangle contour, the height was 300 pixels, and the width was 346.4 pixels subtending a visual angle of 8.25° × 9.5°. When the angles did not disappear toward the target, it would not establish any perception of a subjective contour at all as shown in Figure 4(c). The timing of appearance or disappearance of the sectors was modulated to investigate which factors triggered transient-induced fading.

#### Procedure

Six conditions were randomly presented in one block. Each block was repeated 10 times. Thus, a total of 60 trials (6 conditions × 10 repetitions) were conducted. The general procedure was the same as Experiment 1.

### Results and Discussion

**Figure 5.**
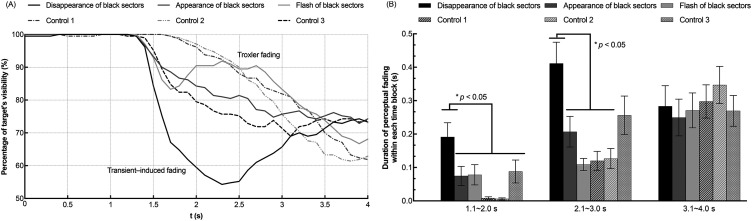
Results of experiment 2. (A) shows the time course results of the target's fading perception. The horizontal axis indicates the time elapsed after stimulus presentation in each trial. The vertical axis indicates the percentage of the target's perceptual visibility. (B) shows the time-blocked results of the target's fading duration in each trial. The horizontal axis indicates the divided three blocks of time course (1.1 ∼ 2.0 s, 2.1 ∼ 3.0 s, and 3.1 ∼ 4.0 s). The vertical axis indicates the duration of the target's fading within each time block. Error bars indicate standard errors of the mean (SEs). The *disappearance of black sectors* (forming a subjective triangle) produced a strong effect on the transient induced fading.

The results of Experiment 2 are shown in Figure 5. The time course results and the time-blocked results are shown in Figure 5(A) and (B), respectively. The method of processing the data was totally the same as Experiment 1.

As shown in Figure 5(A), from 0.1 s to 1.0 s, the target was one hundred percent visible indicating that the target did not fade at all. In the *disappearance of black sectors* condition where the sectors disappeared while the subjective triangle appeared, the target was perceived to fade suddenly at around 1.4 s then gradually became visible again after around 2.3 s. The other five conditions showed a similar tendency where the target gradually faded as time progressed. This indicates that the Troxler fading was primarily induced in the *appearance of black sectors* (disappearance of a subjective triangle), *flash of black sectors*, and the three *control* conditions.

As shown in Figure 5(B), two-way ANOVA revealed that the main effect of the black sector's presentation type (*disappearance, appearance, flash, control 1, control 2,* and *control 3*) was significant for the target visibility [*F*_5, 100 _= 6.524, *p *< .001, η_p_^2 ^= .246]. The main effect of the divided time blocks was also significant for the target visibility [*F*_2, 40 _= 14.403, *p *< .001, η_p_^2 ^= .419]. The interaction between the two independent variables was also significant [*F*_10, 200 _= 8.978, *p *< .001, η_p_^2 ^= .310]. Specifically, multiple comparisons with Bonferroni correction showed that in the time block of 1.1 ∼ 2.0 s, the target's fading was induced longer in the *disappearance of black sectors* (appearance of a subjective triangle) condition than the other five conditions (*p *< .05). In the time block of 2.1 ∼ 3.0 s, except for the *control 3* condition, the target's fading was induced longer in the *disappearance of black sectors* (appearance of a subjective triangle) condition than the other four conditions (*p *< .05).

As a result, the effect of the disappearance of a perceptual object on the transient-induced fading had not been observed because there was no significant difference found between the *appearance of black sectors* (disappearance of a subjective triangle) and the *control* conditions. This contrasts with the *disappearance of black sectors* (appearance of a subjective triangle) condition where a stronger transient-induced fading perception was induced. Taking the results of Experiment 1 into account, the results of Experiment 2 seem to show that the disappearance of local luminance contrast rather than the disappearance of a perceptual object had a greater effect in inducing the fading. However, it should be noted that when the black sectors surrounding the target disappeared (the luminance contrast decrement occurred in the sector areas), the subjective triangle perceptually appeared at the same time. There is a possibility that, besides the luminance contrast decrement, the appearance of a subjective contour may have produced triangular surface representation by filling the new sector color (gray) in the triangular area including the target's area, resulting in the induction of strong perception of transient-induced fading. This is supported by how the transient-induced fading was weaker in the *control 3* condition where the three black sectors disappeared just as in the *disappearance of black sectors* (appearance of a subjective triangle) condition but without composing a subjective triangle. Experiment 3 then thoroughly investigated the effect of subjective contours on transient-induced fading.

## Experiment 3

**Figure 6.**
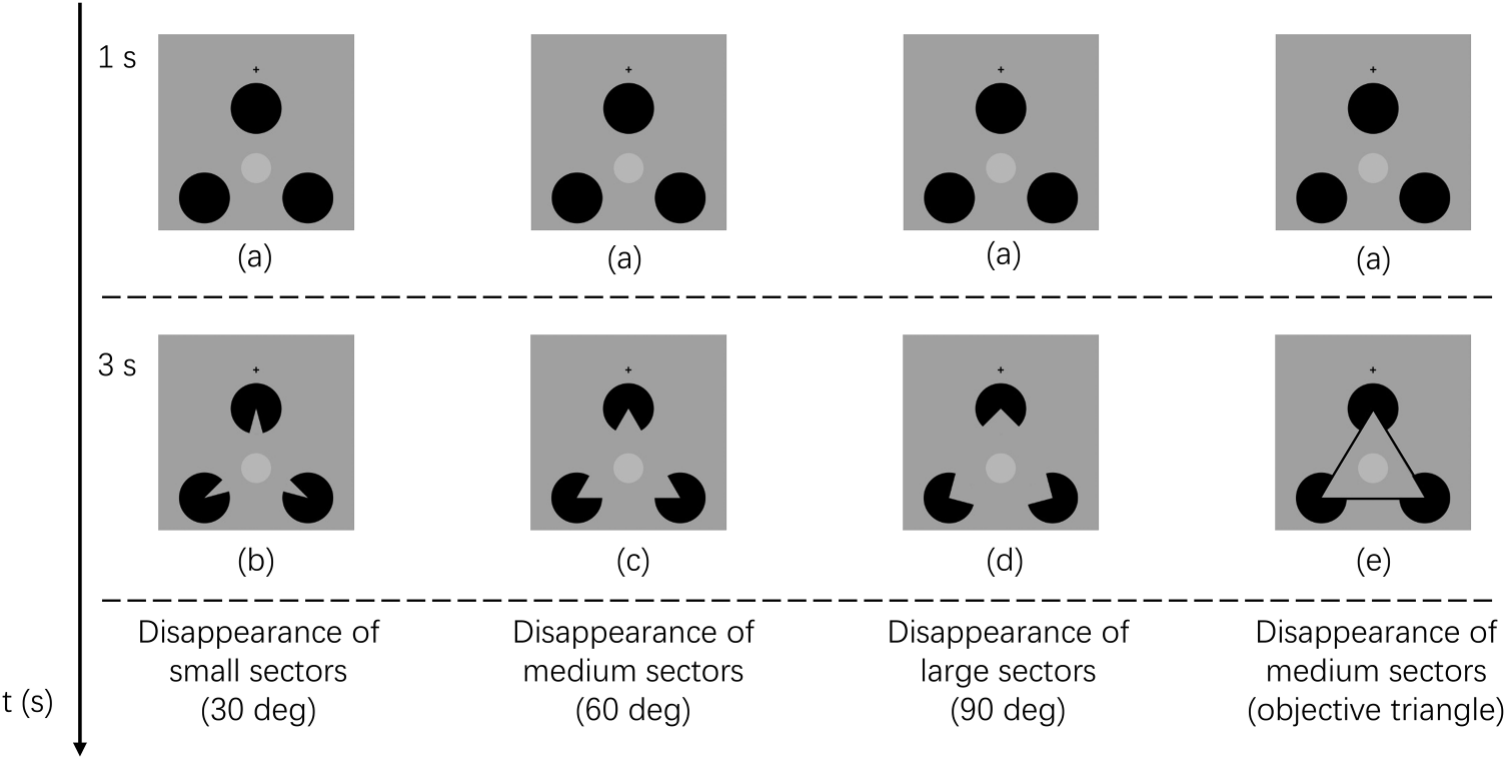
The visual stimulus conditions in experiment 3. The black cross and the target were always presented for 4.0 s throughout all the trials. The stimulus images show the target with (a) three black disks, (b) disappearance of small sectors (30°) forming a subjective contour, (c) disappearance of medium sectors (60°) forming a subjective triangle, (d) disappearance of large sectors (90°) forming a subjective contour, and (e) disappearance of medium sectors (60°) with an objective (physical black) triangle frame. After 1.0 s of presentation, three sectors in black disks disappeared (changed to the same luminance as the background), i.e., a subjective (or objective) contour appeared, for 3 s.

Experiment 3 modulated the area of the black sectors that disappeared to investigate how their disappearance could have an impact on the subjective contour perception and/or transient-induced fading. There were three sector-area disappearance conditions: small, medium, and large sectors, in which the central angles were 30°, 60°, and 90° (Figure 6(b), (c), and (d)), respectively. When the disappearing sector area was medium (i.e., the central angle was 60°), a typical Kanizsa-type subjective triangle contour could be perceived, which was the same as the contour type perceived in Experiment 2. In this condition, it could be expected that the subjective triangle would be perceived most effortlessly (Kanizsa, 1955). Then the subjective triangle perception could induce a filling-in phenomenon which in turn could facilitate the transient-induced fading perception. The large sectors were expected to produce a subjective contour less effortlessly compared to the medium sectors producing the typical subjective contour due to its nonlinear arrangement between the contours of the large sectors. Similarly, the small sectors were expected to produce a subjective contour least effortlessly due to its nonlinearity of sector contours and the smallest disappearance sector size. To confirm this, we first asked the participants to rate how effortlessly (fast and clear) it is to perceive a subjective contour on a six-point scale. We hypothesized that if perceiving a subjective triangle in our stimulus promotes transient-induced fading, a higher rating of subjective contour perception could be related to a stronger transient-induced fading perception. The typical subjective triangle contour (medium sector) condition could induce the strongest transient-induced fading perception in the present study, followed by the large sector, and then the small sector conditions. On the other hand, it could also be assumed that as the disappearing sector size becomes larger, the effect of luminance contrast decrement on transient-induced fading perception could be stronger. The largest sector condition could then induce the strongest transient-induced fading perception, followed by the medium, and then the small sector conditions. By comparing the medium and the large sector conditions, we could test whether the subjective contour perception or luminance contrast decrement was important in inducing fading perception. In addition, a physical black triangle frame was added to the stimulus image in the medium sector condition, shown in Figure 6(e), to compare the effect of subjective contours with that of objective contours on transient-induced fading perception.

### Methods

#### Participants

Twenty-six graduate students (8 males and 18 females) from Kyushu University, participated in this experiment. Among them, three (2 males and 1 female) failed to perceive the target's perceptual fading illusion in the practice session, and consequently they were excluded from the formal session. The age of the twenty-three participants (6 males and 17 females) in the formal session ranged from 21 to 30 years old (24.6 ± 2.2 years). All participants had normal or corrected-to-normal vision and were unaware of the purpose of the study. They were informed prior to the experiment that their privacy was guaranteed, and written consent was obtained from all participants. The study was approved by the local ethics committee of Kyushu University.

#### Apparatus

The apparatus used in Experiment 3 was the same as Experiments 1 and 2.

#### Stimuli

There were four stimulus conditions in Experiment 3 (see Figure 6). The size of the fixation cross and the target (light gray disk) as well as the gray background remained the same as Experiments 1 and 2. The luminance of the target was 94.8 cd/m^2^ and background was 75.4 cd/m^2^. The size of the three black disks used in Experiment 3 remained the same as Experiment 2. The luminance of each black disk was 1.45 cd/m^2^. As with the *disappearance of black sectors* (appearance of a subjective triangle) condition of Experiment 2, each sector in each black disk disappeared after 1.0 s of stimulus presentation. Each disappearing sector was oriented facing the target. The luminance of the sectors when disappearing was the same as the background. The size of the sectors was modulated to be small, medium, or large to make some difference in visibility of the subjective contour, as shown in Figure 6(b), (c) and (d) respectively. When the disappearing sectors were in the medium size, the condition was totally the same as the *disappearance of black sectors* (appearance of a subjective triangle) condition in Experiment 2. As shown in Figure 6(e), a physical black triangle frame was added to the medium (60°) sector condition as an additional stimulus.

#### Procedure

As noted above, before asking the participants if they perceive the transient-induced fading illusion, the participants were first required to rate how effortlessly (fast and clear) they could perceive a subjective contour for small, large, and medium (typical subjective triangle) sectors using a six-point scale. The rating of 0 meant the participant perceived the contour with difficulty or not at all, while 5 meant the participant perceived the contour most effortlessly. The three conditions were randomly presented in one block. Each block was repeated 10 times. Thus, there were total 30 trials (3 conditions × 10 repetitions) for rating subjective contours.

As for the illusion of perceiving the target fading, in Experiment 3, four conditions were randomly presented in one block. Each block was repeated 10 times. Thus, a total of 40 trials (4 conditions × 10 repetitions) were conducted in Experiment 3. Between every five blocks, the participants rested for at least 1 min to eliminate visual fatigue. After resting, they started a new block whenever they were ready to continue.

### Results and Discussion

**Figure 7.**
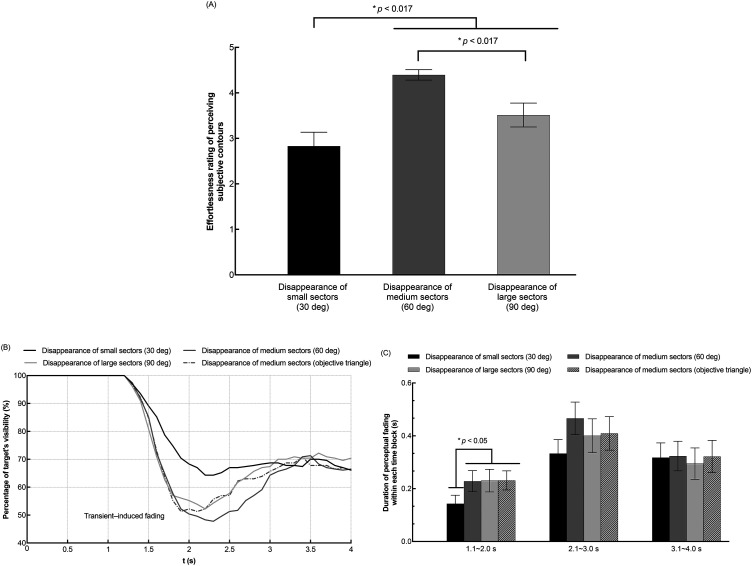
Results of experiment 3. (A) shows the overall “effortlessness” rating for perceiving each subjective contour type. The horizontal axis indicates three subjective contour conditions (small, medium, and large black sectors disappeared). The vertical axis indicates the rating for how effortlessly (fast and clear) it is to perceive a subjective contour (0 = *perceived the contour with difficulty/not at all*, 5 = *perceived the contour most effortlessly*). (B) shows the time course results of the target's fading perception. The horizontal axis indicates the time elapsed after stimulus presentation in each trial. The vertical axis indicates the percentage of the target's perceptual visibility. (C) shows the time-blocked results of the target's fading duration in each trial. The horizontal axis indicates the divided three blocks of time course (1.1 ∼ 2.0 s, 2.1 ∼ 3.0 s, and 3.1 ∼ 4.0 s). The vertical axis indicates the duration of the target's fading within each time block. Error bars indicate standard errors of the mean (SEs).

The results of Experiment 3 are shown in Figure 7. Specifically, Figure 7(A) depicts the rating results showing perceived effortlessness ratings for subjective contours; Figure 7(B) depicts the time course results showing the percentage of target's visibility at each time point as time passed; Figure 7(C) depicts the time-blocked results showing the duration of target's perceptual fading within each time block. As to the percentage of target's visibility in each time point as well as the duration of target's perceptual fading within each time block, the method of data processing was totally the same as Experiments 1 and 2.

As shown in Figure 7(A), Friedman's test showed that there was a statistically significant difference in effortlessness ratings among the three subjective contour types, χ^2^ (2) = 16.00, *p *< .001. Post hoc analysis with Wilcoxon signed-rank tests was conducted with a Bonferroni correction applied, resulting in a significance level set at *p *< .017. Median values (the interquartile range) of perceived effortlessness for the *disappearance of small sectors (30*°*)*, *disappearance of medium sectors (60*°*)* and *disappearance of large sectors (90*°*)* were 2.3 (1.7 to 4), 4.5 (4 to 4.8) and 3.7 (2.5 to 4.8), respectively. Specifically, the effortlessness rating for the *disappearance of small sectors (30*°*)* was lower than the *disappearance of medium sectors (60*°*)* (*Z *= −3.769, *p *< .001) and the *disappearance of large sectors (90*°*)* (*Z *= −2.993, *p *= .003). Meanwhile, the effortlessness rating for the *disappearance of large sectors (90*°*)* was lower than the *disappearance of medium sectors (60*°*)* (*Z *= −2.910, *p *= .004).

As shown in Figure 7(B), from 0.1 s to 1.0 s, the target was one hundred percent visible indicating the target did not fade. The target was perceived to fade suddenly at around 1.2 s in all conditions. The target gradually became visible again at approximately 2.2 s in the *disappearance of small sectors (30*°*)*, the *disappearance of large sectors (90*°*)* and the *objective triangle* conditions; while in the subjective triangle (medium size, 60°) condition it reappeared at around 2.3 s. This indicated that all four conditions produced transient-induced fading.

As shown in Figure 7(C). Two-way ANOVA revealed that the main effect of the contour types was not significant for the target visibility [*F*_3, 66 _= 2.203, *p *= .096, η_p_^2 ^= .091]. The main effect of the time blocks was significant for the target visibility [*F*_2, 44 _= 6.373, *p *= .004, η_p_^2 ^= .225]. The interaction between the two independent variables was not significant [*F*_6, 132 _= 2.077, *p *= .060, η_p_^2 ^= .086]. Although no significant interaction effect was found here, multiple comparisons with Bonferroni correction showed that in the time block of 1.1 ∼ 2.0 s, target's fading duration was shorter in the *disappearance of small sectors (30*°*)* condition than the other three conditions (*p *< .05).

For the 3 subjective contour types (small/medium/large black sectors disappeared), a correlation analysis between the perceived subjective contour ratings and duration of target's perceptual fading within each time block (1.1 ∼ 2.0 s, 2.1 ∼ 3.0 s, and 3.1 ∼ 4.0 s) was also conducted. The Pearson correlation analysis results showed that in the time block of 1.1 ∼ 2.0 s, there was no significant correlation between the subjective contour ratings and target's fading duration in each subjective contour condition (*r *= .028, *p *= .899 in small sector; *r *= −.278, *p *= .199 in medium sector; *r *= −.289, *p *= .182 in large sector). In the time block of 2.1 ∼ 3.0 s, there was also no significant correlation between the factors (*r *= .016, *p *= .942 in small sector; *r *= −.284, *p *= .190 in medium sector; *r *= −.216, *p *= .322 in large sector). Last, in the time block of 3.1 ∼ 4.0 s, there was also no significant correlation found (*r *= −.086, *p *= .696 in small sector; *r *= .129, *p *= .557 in medium sector; *r *= .267, *p *= .217 in large sector).

The results showed that the *disappearance of small sectors (30*°*)* condition had the smallest effect in inducing fading perception compared to the other three contour types. Consistent with our hypothesis, compared with the *disappearance of medium sectors (60*°*)* and *disappearance of large sectors (90*°*)* conditions, the *disappearance of small sectors (30*°*)* condition could lead to a lower luminance contrast decrement as well as a lower subjective contour perception, which could altogether contribute to a weaker transient-induced fading perception. Although the effortlessness rating was higher in the *disappearance of medium sectors (60*°*)* condition than the *disappearance of large sectors (90*°*)* condition, both induced almost the same strong transient-induced fading perception. It could be seemingly assumed that the fast and clear subjective contour perception contributed to the fading perception more than the sector size that disappeared. However, no significant correlation calculated from the 23 participants’ individual data was found between the subjective contour ratings and the target's fading duration in each time block. One possibility could be that the disappearance of the 60° sectors induced the fading effect in saturated strength. This means even though the disappearing sector size was more than 60°, the transient-induced fading perception had reached the ceiling and could not be any stronger.

There was no significant difference of the fading durations between the subjective and objective triangles with 60° vertices as shown in Figure 7(B) and (C). This result additionally supports that the higher visibility of the triangle contour does not necessarily lead to the stronger fading and that the subjective contour does not produce a special fading effect due to subjectivity. Therefore, a hypothesis that filling-in producing the perceptual surface enhances the fading phenomenon is not valid.

## General Discussion

The present study separated two factors (i.e., lower-level factors indicated by the (dis)appearance of the physical changes in local luminance contrasts, and higher-level factors indicated by the (dis)appearance of object-level representation) to investigate which made a greater contribution to transient-induced fading. The present results altogether indicate that the physical changes in local luminance contrast was necessary in inducing strong transient-induced fading. Disappearance of an object at the representation level in itself may not induce the fading phenomenon.

Although the results of Experiment 1 suggested that the offset of the luminance-level inducer (i.e., the black ring) had a stronger effect than its onset, the results of Experiment 2 seem to indicate that the onset of the object-level inducer (i.e., the perceived subjective triangle (Kanizsa, 1955)) also played a crucial role in inducing transient-induced fading. However, it should be noted again that the appearance of a subjective triangle in Experiment 2 was accompanied by the disappearance of the local luminance contrast. Then it would be reasonable to interpret the results as showing that the fading effect was induced not by the object representations, but by the local luminance contrast decrements. In Experiment 3, we tested the remaining possibility that the subjective contour perception also played an important role in inducing transient-induced fading. However, the results showed the higher effortless rating for perceiving a subjective contour did not significantly contribute to a longer target's fading duration.

As to the physical triangle used in Experiment 3, the induced target's fading duration was not different from the subjective contour conditions with the 60° and 90° sectors. This may be explained by the important role of boundary perception (real or illusory) closely linked to perceptual filling-in of surfaces (Weil & Rees, 2011). As Van Lier et al. (2009) described previously, after adapting to the target stimulus, one may perceive that a red or a cyan afterimage fills in the constrained test outlines alternately presented afterward. In addition, Feitosa-Santana et al. (2011) also reported that illusory contours (a Kanizsa square) can bound the filled-in color from a yellow square into an equiluminant achromatic surround in the same manner as real luminance contrast edges. These findings may show some similarity between real and subjective contours in perceptual filling-in. Specifically, for the closed triangle area, both physical black lines and the subjective triangle should block the diffusion and promote the propagation of filling-in signals or surface production. However, we did not acquire any evidence that filling-in or propagation process promotes the target fading.

It is interesting that, while the “appearance” of the subjective contour seems to have induced the fading phenomenon strongly, the three-sector disappearance did not, as found in the *control 3* condition in Experiment 2. Although the same sector areas disappeared in both conditions, the effect was different. If this is not because of the presence of subjective contours, what is the difference between the two stimulus configurations? One possibility is that the three sector areas forming the subjective triangle directly surrounded the target disk while the three sector areas under the *control 3* condition were not oriented toward the target disk. Some previous studies argued that high-level factors such as directing spatial attention to the peripheral target have been shown to increase the probability of perceptual filling-in (De Weerd et al., 2006; Weil & Rees, 2011). Another possibility is that the fading of the target disk may be promoted by a kind of propagation of disappearing signals from the surroundings. The effect of distance between the target and the inducer reported by Kanai and Kamitani (2003) may fit the hypothesis. As Feitosa-Santana et al. (2011) also reported, the larger distance between a chromatic area and the real or illusory contours’ border led to the significantly decreased frequency of perceived filling-in. Thus, the difference between the above two conditions could be tested from three points of view, i.e., attention, spatial configuration of surroundings, and distance between the target and the sectors that have disappeared.

Consistent with prior research (Breitmeyer & Rudd, 1981; Kanai & Kamitani, 2003), the results of Experiment 1 confirmed that a flash stimulus as short as 0.1 s was sufficient to induce a strong transient-induced fading. However, the same fading effect was not observed for the flashed subjective triangle in Experiment 2. The dominancy of disappearing luminance contrast in the fading may provide evidence for why the two *flash* conditions in Experiment 1 and 2 did not induce the same fading effect. Specifically, for the *flash of black ring* condition in Experiment 1, the offset of the inducer was indicated by the disappearance of local luminance contrast (i.e., black ring) resulting in the similar fading effect as the *disappearance of black ring* condition in Experiment 1. For the *flash of black sectors* condition in Experiment 2, the offset of the subjective triangle was indicated by the appearance of three black sectors resulting in the similar Troxler effect as the *appearance of black ring* condition in Experiment 1 as well as the *appearance of black sectors* condition in Experiment 2.

It should be noted that, in Experiment 1, although the *disappearance of black ring* and the *flash of black ring* conditions showed a similar fading tendency, the timings of fading and becoming visible again differed between them. The responses in the *flash of black ring* condition seem to be a little delayed from those in the *disappearance of black ring* condition although we did not find any significant difference between the two conditions as to duration of perceptual fading within each time block (1.1 ∼ 2.0 s, 2.1 ∼ 3.0 s, and 3.1 ∼ 4.0 s). The reason of the delayed response may be explained by the difference in the fading inducing effect between the appearance and disappearance of the black ring in the *flash of black ring* condition. In the *disappearance of black ring* condition, the black ring disappeared at 1.0 s after starting the trial while, in the *flash of black ring* condition, the black ring appeared at 1.0 s and disappeared at 1.1 s. Therefore, the delayed response in the *flash of black ring* condition may be partly explained by the delayed disappearance of the black ring in the *flash of black ring* condition.

As we argued before, Vergeer and van Lier (2007) found the effect of grouping based on similarity. De Weerd et al. (2006) also reported that directing attention to the color, shape, or location of a figure increased the probability of perceiving filling-in compared to unattended figures. What is the difference between our results and theirs? We tried to find the contribution of object representation as an inducer while their study found the effect of similarity grouping of fading targets. Therefore, it may be that “disappearance” in the level of luminance contrast is necessary to trigger the fading as an inducer and it affects the higher-level representation of objects. One may argue that the “appearance” of a subjective contour, which seem to induce the fading phenomenon in Experiment 2, is partially due to the grouping of sector areas that disappeared. At present, we have no evidence to show that the grouping of inducers, not just surrounding a target, enhanced the illusion. This hypothesis that the grouping effect, apart from forming an illusory contour promotes the target fading may be worth testing.

Through the three experiments, we tested which type of (dis)appearance induced the transient-induced fading, (dis)appearance in luminance contrast or (dis)appearance of a perceptual object. We conclude that the disappearance of areas with high luminance contrast surrounding the target is necessary for the perceptual fading. The fading phenomenon may be triggered by a low-level signal, although higher-level grouping factors as observed in Vergeer and van Lier (2007) may also have an influence on determination of objects that fade away. Our findings revealed that, in the transient-induced fading paradigm, different levels of processing are involved with the trigger and its result (fading), which may provide a key idea of research in other fading phenomena such as Troxler fading or contrast decrement disappearance (May et al., 2003).
